# Gender Differences in Experiences of Leadership Emergence Among Emergency Medicine Department Chairs

**DOI:** 10.1001/jamanetworkopen.2022.1860

**Published:** 2022-03-10

**Authors:** Cherri Hobgood, Claire Draucker

**Affiliations:** 1 Emergency Medicine at Indiana University School of Medicine, Indianapolis; 2 Angela Barron McBride Professor of Psychiatric Nursing at Indiana University School of Nursing, Indianapolis

## Abstract

**Importance:**

The number of women entering medicine continues to increase, but women remain underrepresented at all tiers of academic rank and chair leadership in EM. The proportion of female chairs in EM has not exceeded 12% in 2 decades.

**Objective:**

To compare how male and female EM chairs experience leadership emergence, with attention to factors associated with support of the emergence of female chairs.

**Design, Setting, and Participants:**

This qualitative descriptive study was conducted between April 2020 and February 2021 at 36 US academic EM departments. Eligible participants were all current and emeritus female EM academic department chairs (with a possible cohort of 20 individuals) and an equal number of randomly selected male chairs.

**Interventions:**

Semistructured interviews were conducted via teleconferencing with an 11-item interview guide.

**Main Outcomes and Measures:**

Qualitative findings identifying similarities and gender differences in leadership emergence were collected.

**Results:**

Among 20 female chairs in EM, 19 women (mean [SD] age, 56.2 [7.1] years) participated in the study (95.0% response rate). There were 13 active chairs, and 6 women were within 5 years of chair leadership. Among 77 male chairs in EM identified and randomized, 37 men were invited to participate, among whom 19 individuals (51.4%) agreed to participate; 18 men (mean [SD] age, 52.2 [7.5] years) completed their interviews. Reflecting upon their experiences of leadership emergence, male chairs saw leadership as their destiny, were motivated to be chairs to gain influence, were dismissive of risks associated with chairing a department, and were sponsored by senior male leaders to advance in leadership. Female chairs saw leadership as something they had long prepared for, were motivated to be chairs to make a difference, were cautious of risks associated with chairing a department that could derail their careers, and relied on their own efforts to advance in leadership.

**Conclusions and Relevance:**

This study found that experiences of leadership emergence differed by gender. These results suggest that leadership development strategies tailored to women should promote early internalization of leadership identity, tightly link leadership to purpose, cultivate active sponsorship, and encourage women’s risk tolerance through leadership validation to support women’s development as leaders and demonstrate a commitment to gender equity in EM leadership.

## Key Points

**Question:**

How do male and female emergency medicine (EM) department chairs emerge as leaders, and are there gendered differences in their experiences?

**Findings:**

This qualitative study of leadership in 18 male and 19 female EM department chairs identified gender differences in the following areas: identity as leaders, motivations to become chairs, considering the risks of being chair, and leadership advancement.

**Meaning:**

These findings suggest that women’s leadership may be fostered by transparency of purpose, strong sponsorship by senior leaders, and the creation of safe leadership environments for women within academic medicine.

## Teaser

This qualitative study examines differences in the experience of leadership emergence among men and women emergency department chairs.

## Introduction

Emergency medicine (EM) has experienced unprecedented growth over the last 2 decades, but gender parity of its leaders has not evolved. Since 2001, US academic departments of EM have nearly doubled (from 60 departments in 2001^1^ to 114 departments in 2020^2^) and EM residency has become a top 5 specialty choice.^3^ Women, now making up 50% of medical students,^4^ comprise 37% of EM trainees^5^ and 38% of EM faculty.^6^ However, the proportion of women holding department chair positions in EM remains between 10% (2001)^7^ and 11.3% (2020).^2^

The long-standing belief that increasing the number of women in medicine would lead to more women leaders has been proven false.^8^ Women in medicine publish less,^9^ are less successful in obtaining long-term federal funding,^10^ and lag in faculty rank progression.^5,11^ Women faculty lack academic rank parity, and as rank increases, women’s rank parity declines.^12,13^ Moreover, women who assume leadership positions are more likely to become assistant or associate deans of education, faculty development, and diversity than department chairs.^5^ At the highest tier, however, fewer than one-third of center or institute directors and 18% of academic department chairs and medical school deans are women.^5^

In EM, women faculty complete more subspecialty fellowship training and EM female chairs hold more advanced degrees, extramural funding, and national organization leadership positions than their male colleagues.^14^ However, women in EM remain at disproportionately lower tiers of rank and pay for all ranks and quartiles of clinical load.^13,15^ Despite these disparities, little is known about how women in EM become leaders or how their experiences differ from those of their male counterparts. The purpose of this study was to compare how male and female academic department chairs in EM experience leadership emergence, with attention to the factors associated with support for the emergence of female chairs.

## Methods

The institutional review boards of Indiana University and the University of North Carolina schools of medicine approved the protocol for this qualitative study. Study details were disclosed, and verbal consent was obtained. We followed the Consolidated Criteria for Reporting Qualitative Research (COREQ) reporting guideline.

### Design

A qualitative descriptive study^16^ with a cohort comparison group was conducted. Qualitative description is used when the researcher seeks to describe a phenomenon in the everyday terms of participants. The product of qualitative description is a straightforward but comprehensive summary of narrative text that can answer pragmatic questions of interest to practitioners and policy makers. Purposive sampling, semistructured interviews, and content analysis are procedures most frequently used for this method. This method was chosen for this study given that the aim was to provide a straightforward description of how male and female EM department chairs emerge as leaders and to examine gendered differences in their experiences.

### Sampling and Recruitment

The all-woman research team (see acknowledgment section) completed a personal positionality memo before exposure to study protocols or data. Purposive criterion-based sampling was used to identify academic EM department chairs. In 2019, the Association of American Medical Colleges identified 13 female and 101 male EM department chairs.^17^ To increase the participant pool, criteria for women were expanded to include emeritus chairs within 5 years of role completion. Participants were identified by cross-referencing the Association of Academic Chairs in Emergency Medicine (AACEM) listserve participants and AACEM chair development rosters with university websites. All eligible women leaders were invited to participate. A roster of male chairs was similarly developed and randomized using Research Randomizer, (Social Psychology Network).^18^ Male chairs were sequentially invited until the study groups were balanced in size. Potential participants received personalized emails describing the study and inviting participation. Nonresponders received 2 email reminders.

Interviews were held via recorded Zoom videoconference (Zoom Video Communications) sessions. A single interviewer (C.H.) conducted individual interviews between April 2020 and February 2021. Based on literature review and knowledge of the discipline, an 11-item interview guide had been developed, piloted, and iterated for question clarity (eAppendix in the Supplement). The interview guide structured the interview, but participant responses were unrestricted and determined the flow of discussions. Data were securely recorded, deidentified, and transcribed. Participants self-reported demographic information.

### Statistical Analysis

Demographic data were summarized using descriptive statistics, including means and SDs, medians and IQRs, frequencies, and percents. Qualitative data was inductively coded using NVivo software version 12 (QSR International). Two coders independently reviewed a 9-transcript subsample and coded units of text related to study aims. An initial coding framework was defined by team discussion using comparison and consensus. Using this framework, team members independently coded remaining transcripts. If new codes emerged, or existing codes required clarification, the framework was collaboratively reviewed and iterated. Among transcripts, 15% were double-coded and compared using κ coefficients with text characters as the unit of comparison. Team consensus was used to review and resolve all areas of disagreement or κ < 0.6.

For this report, codes related to study aims were extracted, transcript data were reexamined, and codes were iteratively grouped into themes. Within themes, gendered differences were identified. A researcher (C.H.) prepared a textual summary of findings, and other team members confirmed narratives. Member checks were performed by sharing findings, supported by deidentified quotes, with participants for verification.

## Results

### Demographics

Among 20 women who met study criteria, 19 women (mean [SD] age, 56.2 [7.1] years) agreed to participate and were enrolled (95.0% response rate). There were 13 active chairs, and 6 women were within 5 years of chair leadership. Among 77 eligible male chairs identified, 37 men were invited to participate; 19 agreed to participate and were enrolled (51.4% response rate). One scheduled subject did not attend his interview, yielding a final sample of 18 male chairs (mean [SD] age, 52.2 [7.5] years). Participants represented 36 academic departments of EM. Table 1 presents participant demographics and information about prior leadership experiences.

**Table 1.** Demographic Characteristics by Gender

Characteristic	Emergency medicine chairs, No. (%) (N = 37)	
	Women (n = 19)	Men (n = 18)
Institutions, No.	18	18
Age, y		
Mean (SD)	56.2 (7.1)	52.2 (7.5)
Median (IQR)	57.0 (49.0-60.0)	50.5 (47.0-59.3)
Time in chair role, y		
Range	2.0-12.0	0.8-19.0
Mean (SD)	6.5 (3.5)	7.2 (5.1)
Median (IQR)	6.0 (4.0-11.0)	6.5 (3.8-9.6)
Role preparation		
Additional degrees^a^	12 (63.2)	4 (22.2)
Prior leadership roles		
UME^b^	12 (63.2)	4 (22.2)
GME^c^	15 (78.9)	10 (55.6)
UME and GME	11 (57.9)	2 (11.1)
Research^d^	12 (63.2)	6 (33.3)
Operations^e^	9 (47.4)	7 (38.9)
Dean’s office^f^	5 (26.3)	2 (11.1)
Vice chair	5 (26.3)	9 (50.0)
Interim, acting, or EM division chief^g^	8 (42.1)	8 (44.4)
National board service^h^	9 (47.4)	5 (27.8)
Inaugural chair	7 (36.8)	4 (22.2)
Actively sought role^i^	13 (68.4)	16 (88.9)

Abbreviations: EM, emergency medicine; GME, graduate medical education; UME, undergraduate medical education.

^a^ Additional degrees include MPH, MS, MSC, and MBA.

^b^ UME includes any service as clerkship director, course director, and vice chair of education.

^c^ GME includes any service as assistant or associate program director, program director, or vice chair of education.

^d^ Research includes any service as research director, division chief, vice chair of research, or extramural funding.

^e^ Operations includes any service as medical director, vice chair of operations, or service line chief.

^f^ Dean’s office includes any service as assistant or associate dean–titled role.

^g^ Represents full Division of Emergency Medicine in Department of Surgery or Internal Medicine and includes serving as division chief, acting, or interim full department or section leadership.

^h^ National board service includes service in any of the following organizations: American Academy of Emergency Medicine, Association of Academic Chairs of Emergency Medicine, American Board of Emergency Medicine, American College of Emergency Physicians, Accreditation Council for Graduate Medical Education-Residency Review Committee, American College of Osteopathic Emergency Physicians, Council of Residency Directors in Emergency Medicine, Society for Academic Emergency Medicine, and National Association of EMS Physicians.

^i^ Actively sought role indicates participant applied for the position rather than was appointed without a process.

### Qualitative Findings

Chairs discussed a variety of experiences associated with their emergence as department chairs. Participants of both genders enjoyed personal satisfaction by building programs, improving systems, and “growing” faculty. However, their leadership emergence experiences differed in 4 areas: identity as leaders, motivation to become chairs, considering the risks of being chair, and leadership advancement. Table 2 depicts these 4 areas and includes supporting verbatim quotes.

**Table 2.** Gender Differences in Experiences of Leadership Emergence

Areas of gender difference	Exemplar quotes	
	Male chairs	Female chairs
Identity as leader	Destiny: “I can't say I was a good grade school athlete because I was a little short guy, but I wanted to be a leader in sports. And so in high school, I wound up being a captain of both the soccer team and the swimming team. Same thing going into college. My goal was to be the captain of the rowing team. That kind of stuff just carried through.” —participant M-12	Preparation: “I actively looked for opportunities to contribute, and it's hard work, right? We know it's hard work. It takes time, but I personally feel like it's [leadership] an obligation.” —participant F-19
Motivation to become chair	Gaining influence: “When I started, we had no one in any senior leadership within the organization. I've been very strategic and really tried to identify openings and recruit specifically to fill those positions. It's not by accident, I think, emergency medicine has become much more influential in the health system and the school.” —participant M-14	Making things better: “It was an opportunity to build something from the ground-up, which inspired me. I saw an opportunity to make change and make a difference, and that's what drives me.” —participant F-15
Considering risks of being chair	Dismissive: “I simply made the assessment that the risk balance of becoming the chair for me, personally, was better, more favorable than not being the chair.” —participant M-16	Cautious: “The real risk is that it’s almost as if women are disposable leaders. They put us in on the most difficult jobs, with the biggest issues, and if you do a great job, that's great, but ultimately if you fail or if you have a misstep, you know, you get one, and your career is over.” —participant F-11
Leadership advancement	Sponsored: “He knew I wanted to be the residency director, and he helped get me there. And then, he started to talk to me about being chair, and he created a vice-chair position that would promote me, to get me ready.” —participant M-7	Self-directed: “I chose this title and have worked for it for my whole life. I became a leader to do these things and help people get where they're going while still achieving the goals of the organization.” —participant F-17

### Identity as Leaders: Destiny vs Preparation

Male chairs typically saw leadership as their legacy. However, female chairs identified leadership as something for which they had long prepared.

#### Male Chairs

Early on in their careers, male chairs viewed themselves as leaders and revealed that becoming a chair was part of a career journey that was destined. They felt that they were ordained to become leaders and could “see” themselves as chairs. Many recounted leadership roles in high school and college, especially in sports. Participant M-14 said, “I think, to a certain extent, people who are leaders, it’s a little bit in their DNA, and it’s a little bit trained. I mean, it’s something that, you know. I was a captain of my wrestling team in high school, and I started taking small leadership roles early on in my career.” Some male participants had little preparation to become chairs but believed in their natural abilities to successfully lead. Moreover, because they believed leadership was their fate, several participants assumed that they would advance to even higher leadership positions.

#### Female Chairs

Female chairs viewed leadership as something for which they had long prepared. All participants had actively sought growth and leadership opportunities throughout their careers. Some participants considered becoming chairs the next logical step, whereas others were ambivalent and considered themselves “accidental” chairs. Many participants required coaxing by senior leaders to accept chair roles and were pressed into leadership because of their skills. They were often needed to fix departmental problems, salvage floundering programs, or calm difficult interpersonal situations. Participant F-1 said, “I was someone who always liked being involved but not necessarily being the lead captain. When our dean told me, ‘You know, when you talk, they listen; you have their alliance,’ that's when I stepped back and said, ‘OK, I really care about this. I care about my colleagues. I care about my nurses. I care about residency training, and so I'm going to go ahead and take this role.’”

### Motivations to Become Chair: Gaining Influence vs Making Things Better

Male chairs typically focused on the opportunity to increase their influence and that of EM. Meanwhile, female chairs typically focused on the opportunity to make things better in their departments.

#### Male Chairs

Upon assuming their roles, male chairs sought to make their departments “great” and thereby gain influence within their institutions and in the medical community at large. Several participants saw building strong departments as a way of increasing the prestige of EM in academic and practice communities, as well as of advancing their own reputations. Male participants said they wanted their success as chair to be “my stamp” or “part of my legacy.” Participant M-13 said, “I am either going to build something that I can be proud of and will be the envy of a lot of people—because, I mean, there's ego involved in all this—or I’ll go do something else. I'm still pretty young. You have to be unapologetic about making it great, to create a vision that smart people want to buy into so they want to come and work for you.”

#### Female Chairs

Female chairs sought to generate positive change in their departments rather than to seek influence. Participant F-13 said, “When I was asked to become the interim [chair], I declined. Then, I started thinking about what’s important for me. I don’t just want to hold the title; it’s very important to me to make a difference.” Female chairs sought to be transformative leaders because they were inspired by the untapped potential of the institution, their department, and the faculty. Whether they ascended to the chair role from inside or outside the institution, they were driven to make things better. Participant F-6 said, “When the position [chair] came open, it felt like a unique opportunity to step in and to lead the department forward by maximizing the potential that existed within it. That's how I got here. There was a need. There was an opportunity for me to mentor and nurture and grow and develop, and so I stepped forward.”

### Considering the Risks of Being Chair: Dismissive vs Cautious

Male chairs typically dismissed risks associated with assuming a chair position as being expected. However, female chairs were cautious about these risks and concerned about how such risks would influence their leadership.

#### Male Chairs

Male chairs acknowledged the risk of failure that accompanied taking on a chair role but typically felt “comfortable” with the risks. They identified risk as inherent in any transition and dismissed it as an important consideration in taking on the chair role. Some participants stressed that all success comes with risk. They indicated that if they failed, they would move on but would not let risk interfere with taking the chair position. Participant M-7 said, “If I did it and I failed, or if I do, that'll be very hard. I'll have to figure out how to get around that. But the idea of not doing it [becoming chair] or sitting on the sideline, I don't think I could live with that.”

#### Female Chairs

Female chairs also acknowledged the risk of failure that accompanied taking on the chair role but were more cautious than their male counterparts. Many female participants were aware that the departments over which they were assuming leadership were plagued with problems that they would be expected to solve. They worried that the extent of these problems made them uniquely vulnerable to failure. Unlike their male counterparts, who suggested that they would just “move on” if they failed, female chairs believed failure would disadvantage their careers long term. Female chairs also believed that women leaders tend to pay a higher price for failure and that institutions should provide a “culture of safety” for women leaders. Participant F-6 said, “Institutions need to create a safe environment in which women can explore. Go to a leadership gym and exercise some leadership muscle. We shouldn’t blame the individual when they fail. We should look at systemic issues involved, then try to rectify those to create an environment in which people don't struggle.”

### Leadership Advancement: Sponsored vs Self-directed

Male chairs were typically sponsored by senior leaders who provided advancement support and opportunities. Female chairs, however, typically advanced through their own hard work and effort.

#### Male Chairs

Male chairs indicated that their leadership advancement was enhanced by sponsorship of senior leaders, especially senior male physicians. These champions guided male chairs, validated their leadership, and nominated them for institutional and national leadership opportunities. These relationships “fast-tracked” these participants’ careers. Male chairs leveraged these relationships and thus acquired powerful allies and strong professional networks. Participant M-18 explained, “We [he and a senior colleague] developed a very strong relationship over 3 or 4 years. And when he became the CEO, he asked me to take his current role, which was not something I had aspired to. It was quite surprising, but I leapt at the opportunity because I really love the guy and he had been a mentor for me over the prior 4 or 5 years.”

#### Female Chairs

Female chairs indicated that their advancement in leadership was primarily because of their own efforts. In contrast to their male counterparts, they were rarely championed by senior colleagues. Instead, they worked hard, showed investment in their work, took on a variety of responsibilities, and were dedicated to their goals. Participant F-16 said, “I’ve ended up in leadership positions because I did things. You know, it's like I showed up. I invested my time and, quite honestly, worked very hard.”

## Discussion

This qualitative study found that male and female EM chairs did the same work; they did strategic planning, built programs, improved systems, and empowered faculty. They differed, however, in why they chose to lead and how their leadership emerged.

Our findings resonate with prior work on women in leadership. The finding that female chairs emphasized purpose rather than personal influence, for example, is consistent with reports finding that sense of purpose is associated with women leaders, especially in cultures that emphasize masculine ideals, retaining authenticity, assuming greater social risk, and internalizing a leadership identity.^19,20,21^ Moreover, the finding that male chairs enjoyed sponsorship by senior colleagues, whereas this sponsorship was less available to female chairs, has been reflected in prior literature. Several reports have acknowledged the importance of sponsorship within academic medicine.^22,23^ In surgery, for example, informal sponsorship is cited as a critical element in the success of women department chairs.^24^ External endorsement from a powerful organizational sponsor is associated with fortified leadership potential in a protégé, the building of reputational capital, and increased risk tolerance, all of which legitimize leadership.^19,22,25^ In business, essential organizational knowledge is transmitted through sponsorship networks, and the lack of networks is an acknowledged barrier to women’s leadership advancement.^26,27,28^

Our findings, supported by prior literature, may provide actionable guidance to develop strategies to support women’s movement into leadership and to increase gender parity in EM and across academic medicine. First, our findings suggest that strategies should be developed to increase rates of sponsorship for women in academic medicine.^22,23,25^ Some experts have suggested that women’s risk intolerance and role insecurity may inadvertently signal distrust in sponsors’ judgements and derail their willingness to sponsor a woman, instead seeking “rising stars” who remind them of themselves.^22,25^ This type of homosocial reproduction is associated with reinforced gender imbalances and reproduction of status quo occupational power inequities.^20,29^ To counteract this phenomenon, a formalization of sponsorship opportunities may be provided for women leaders in academic medicine to enhance equity in opportunities. Formal sponsor relationships should support female chairs in their leadership roles, validate and advance their leadership skills, and provide advancement opportunities within the institution and profession.

In addition, our findings suggest that academic medicine should foster and reward rather than dismiss women leaders’ sense of purpose or commitment to make positive change in their departments rather than to seek personal influence or prestige. Because women leaders often take over departments that are problem ridden, they should be provided with human and material resources they need to meet department goals, as well as leeway to take risks and at times fail without experiencing disadvantage to their career advancement.

Finally, this research suggests that systemic institutional factors that hinder women’s leadership advancement should be examined. For example, institutional practices in which women are encouraged to assume a variety of leadership responsibilities that are not rewarded or are required to acquire additional credentials to demonstrate their competence or affirm their leadership should be challenged.^19,20,30,31^ With an institutional climate that support women’s advancement in academic medicine, women may be more likely see themselves as destined for leadership.

### Limitations

This study has several limitations. It was limited by small sample size, constrained by the small number of female EM chairs. However, in-depth descriptions and abundant examples of leadership experiences from male and female chairs allowed us to address study aims. The gender and prior experiences of the research team may have introduced bias; however, a priori positionality statements and task segregation, such as using nonclinician coders for primary data analysis, theme verification, and member checks, may have served to counterbalance this influence. Interviews were conducted by 1 team member (CH) who is a woman and former EM chair. While this may have introduced some bias, it also provided a shared understanding between the participants and interviewer that may have motivated participants to disclose information they may not otherwise have shared. The small number and lower response rate among male chairs may have generated a nonrepresentative sample limiting our ability to make broad generalizations about male leadership emergence.

## Conclusions

This study found that women in EM who emerged as leaders were motivated by purpose and inspired by the opportunity to make meaningful change. Our findings suggest that creating an alignment of purpose coupled with early leadership opportunities and strong sponsorship may be associated with greater emergence of women leaders. Advancing women as leaders will require intentionality and the commitment of institutional and discipline leaders to mitigate factors associated with delegitimization of women’s leadership and create environments that foster their unique leadership skills.

## Notes

Supplement. **eAppendix.** Semistructured Interview Guide for Male and Female Emergency Medicine Department Chairs
